# Real-World Prescribing Patterns of Clomiphene Citrate for Male Infertility: A National Cross-Sectional Survey of Urologists in Türkiye

**DOI:** 10.3390/jcm15135014

**Published:** 2026-06-27

**Authors:** Tuncer Bahceci, Gökhan Çeker, Erman Ceyhan, Ali Can Albaz, Mesut Berkan Duran, Cevahir Özer, Murat Gül

**Affiliations:** 1 Department of Urology, Faculty of Medicine, Aydin Adnan Menderes University, 09970 Aydin, Türkiye; 2 Department of Urology, Başakşehir Çam and Sakura City Hospital, 34480 Istanbul, Türkiye; drgokhanceker@gmail.com; 3 Department of Urology, Faculty of Medicine, Baskent University, 06490 Ankara, Türkiye; erman_ceyhan@yahoo.com; 4 Department of Urology, Faculty of Medicine, Manisa Celal Bayar University, 45140 Manisa, Türkiye; alicanalbaz@hotmail.com; 5 Department of Urology, Faculty of Medicine, Pamukkale University, 20160 Denizli, Türkiye; drberkanduran@gmail.com; 6 Department of Urology, Faculty of Medicine, Baskent University, 01250 Adana, Türkiye; cevahirozer@baskent.edu.tr; 7 Department of Urology, Faculty of Medicine, Selcuk University, 42130 Konya, Türkiye; murat.gul@selcuk.edu.tr

**Keywords:** clomiphene citrate, male infertility, physician practice patterns

## Abstract

**Background/Objectives:** Clomiphene citrate (CC) is widely used off-label for male infertility despite limited evidence and inconsistent guideline recommendations. Although previous studies suggest variability in clinical practice, real-world data on prescribing patterns, patient selection, monitoring, and treatment success definitions remain limited. This study assessed CC prescribing patterns among urologists and identified factors associated with its use. **Methods:** A national, cross-sectional, web-based survey was conducted among urologists in Türkiye between November and December 2025. Of 1558 invited participants, 421 responded (27.0%), and 402 were included in the final analysis. The questionnaire was based on European Association of Urology and American Urological Association guidelines, refined through expert consensus, and pilot-tested. Multivariable logistic regression identified factors independently associated with CC use. **Results:** CC was used by 39.3% of respondents and was independently associated with private practice (odds ratio [OR] = 2.90, *p* < 0.001), greater professional experience (OR = 2.18, *p* = 0.002), and higher infertility case volume (OR = 2.27, *p* = 0.001). Substantial heterogeneity was observed in patient selection, dosing, monitoring, and success definitions. Treatment goals and perceived success definitions most frequently focused on laboratory-based endpoints, including semen parameters and testosterone levels, which were more frequently selected than pregnancy-related endpoints. However, spontaneous pregnancy was also commonly reported as a perceived success definition, whereas live birth was not separately assessed. An apparent indication paradox was observed for hypogonadotropic hypogonadism, which may reflect differing interpretations of functional versus irreversible hypogonadotropic states, and 31.6% of clinicians reported not routinely providing risk counseling. **Conclusions:** CC prescribing for male infertility remains heterogeneous among responding urologists and was associated with clinician experience, practice setting, and infertility case volume rather than standardized protocols. The predominance of laboratory-based endpoints, together with the frequent inclusion of spontaneous pregnancy as a perceived success definition and the absence of separate live-birth assessment, underscores the need for clearer terminology, standardized prescribing frameworks, structured risk counseling, and future studies incorporating clinically meaningful reproductive endpoints.

## 1. Introduction

Infertility affects a significant number of couples worldwide and is defined as the inability to conceive after 12 months of regular unprotected intercourse [1,2]. Male factors account for about 40% of cases, highlighting the importance of male reproductive health in fertility treatment [3]. Despite advances in diagnostic methods, many men are still diagnosed with idiopathic infertility, for which proven treatment options are limited [4,5,6,7,8].

In this context, empirical medical therapies (EMT), including antioxidants and hormonal agents, are often used despite limited supporting evidence. Among these, clomiphene citrate (CC), a selective estrogen receptor modulator (SERM), is frequently prescribed off-label for men to stimulate endogenous gonadotropin and testosterone production [9,10]. Although generally well tolerated, its clinical application shows significant variability in dosing, treatment duration, patient selection, and outcome assessment [9,11,12,13,14].

Current international guidelines emphasize the low quality of evidence supporting the use of SERMs in male infertility and avoid making strong recommendations [4,8]. Similarly, recent meta-analyses indicate that although CC may improve surrogate outcomes, such as semen parameters and hormonal profiles, its impact on clinically meaningful endpoints, such as pregnancy and live birth, remains uncertain [15,16]. Despite this uncertainty, survey data indicate that CC remains widely used in clinical practice, highlighting a gap between guideline recommendations and actual practice [17,18,19]. However, detailed real-world data on prescribing patterns, monitoring strategies, and definitions of treatment success are still limited, especially at the national level.

To our knowledge, this is among the first national cross-sectional surveys among responding urologists to examine real-world prescribing patterns of CC. The study highlights a critical gap between guideline recommendations and routine clinical practice by offering insights into prescribing behavior, patient selection, monitoring strategies, and outcome assessment. By characterizing these patterns and evaluating perceived effectiveness and safety, the findings could inform future guideline development, support the standardization of care, and align clinical decision-making with patient-centered fertility outcomes.

## 2. Materials and Methods

### 2.1. Study Design and Ethical Approval

This cross-sectional, web-based survey aimed to assess current clinical practices among urologists in Türkiye regarding the use of CC in managing male infertility. The study protocol received approval from the Ethics Committee of Aydın Adnan Menderes University (Approval No: 2025/298). Participation was voluntary and anonymous, and electronic informed consent was obtained from all participants before the survey commenced.

### 2.2. Survey Development and Structure

Clomiphene citrate (CC) was selected as the focus of this study because it is widely used as empirical medical therapy for male infertility and serves as a pragmatic model for examining real-world prescribing behaviors [17,20]. Although other empirical treatments, such as aromatase inhibitors and gonadotropins, are also used in selected clinical settings, CC’s widespread off-label use and variability in prescribing practices make it particularly suitable for evaluating patterns of clinical decision-making at a national level [17,18,19].

The questionnaire was developed by the corresponding author based on current European Association of Urology (EAU) and American Urological Association (AUA) guidelines [4,8] and previously published international surveys [17,18,19]. The initial draft was reviewed by seven urologists with expertise in andrology and male infertility, all members of the Andrology Working Group of the Society of Urological Surgery in Türkiye [21]. Based on their feedback, the questionnaire was revised. A structured online consensus meeting was then conducted, during which all items were reviewed and finalized. The revised questionnaire was pilot-tested with a separate group of 10 urologists to assess clarity, understanding, and practicality. The final version was implemented using Google Forms (Google LLC, Mountain View, CA, USA), with all items marked as mandatory to ensure complete responses. Although the questionnaire underwent expert review, consensus development, and pilot testing to support content validity, formal psychometric validation was not performed, as the tool was designed to capture observable clinical behaviors and practice patterns rather than latent constructs [22,23].

The final questionnaire included three main sections: (1) demographic and professional characteristics; (2) reasons for not using CC among non-users; and (3) detailed clinical practices among CC users, such as patient selection, indications and contraindications, dosing strategies, monitoring approaches, treatment goals, perceived treatment success, and adverse effect management.

CC user status was determined using the following screening item: “Do you use clomiphene citrate treatment for male infertility in your routine clinical practice?” Response options were “Yes” and “No.” Respondents who answered “Yes” were classified as CC users, whereas those who answered “No” were classified as non-users. This variable reflected self-reported routine clinical practice at the time of survey completion, rather than lifetime or historical use. The screening item did not distinguish between occasional and systematic prescribing; subsequent items among CC users collected further details regarding indications, dosing, monitoring, and treatment goals.

Participants classified as CC users answered 28 items, while non-users completed a shorter 10-item version.

Likert-scale items were used to assess the perceived frequency of adverse events, and an optional open-ended question allowed participants to provide additional comments.

### 2.3. Data Collection and Study Population

The survey invitation was distributed through national and regional WhatsApp groups affiliated with the Society of Urological Surgery, including regional branch groups covering all seven geographical regions of Türkiye. However, participants’ individual city or region of practice was not collected. Members were invited twice: an initial invitation was sent in November 2025, and a reminder message was sent one month later to enhance participation. The survey was open from November 2025 to December 2025.

**Figure 1:**
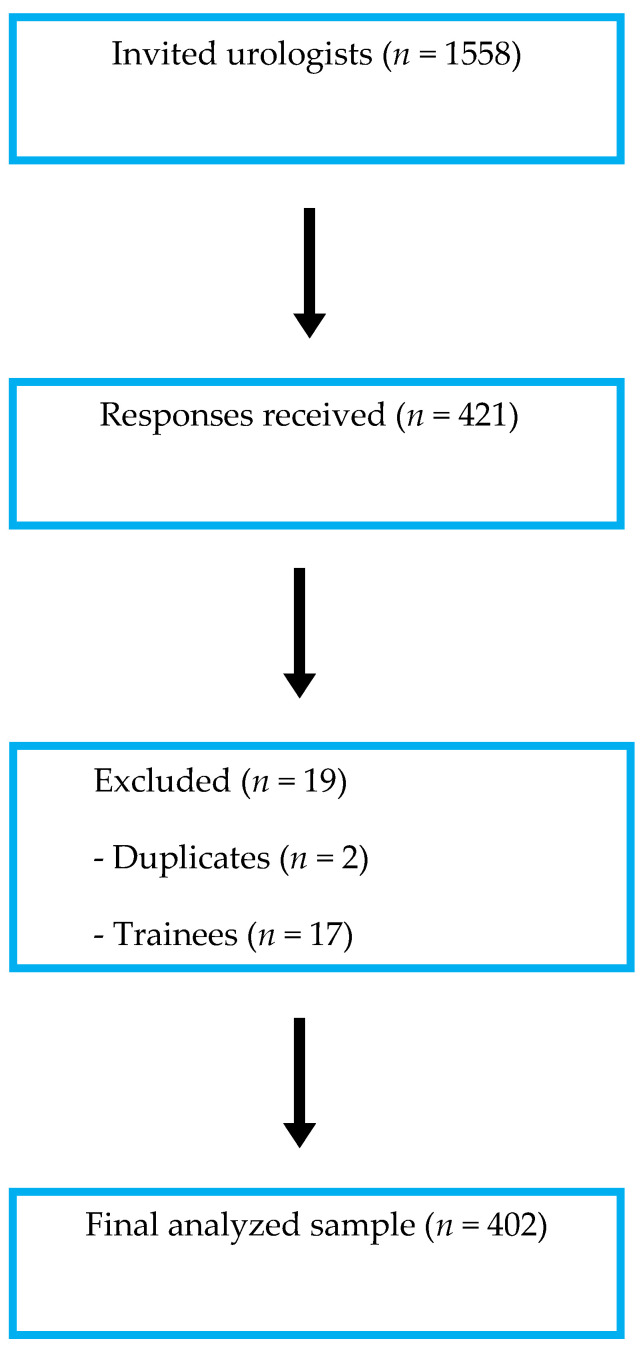
Flow diagram of survey dissemination, responses, exclusions, and final analytic sample.

The invitation was sent to about 1558 urologists. A total of 421 responses were received (response rate: 27.0%). After excluding responses from residents and duplicate submissions, 402 responses were included in the final analysis; the participant flow is shown in Figure 1.

In accordance with key CHERRIES recommendations, additional web-survey procedures were specified. The survey was anonymous and did not collect identifying information, IP addresses, cookies, or login-based identifiers. Therefore, technical prevention of multiple submissions through IP- or cookie-based restrictions was not applied. Duplicate responses were screened after data collection by reviewing submission timestamps together with identical demographic and professional response patterns; two duplicate submissions were identified and excluded. The number of invited urologists was estimated from the membership of the professional WhatsApp groups used for dissemination; however, the number of individuals who actually viewed or opened the invitation could not be determined because the recruitment platform did not provide reliable view/open tracking. Adaptive questioning was used: respondents who answered “Yes” to the CC-use screening item were directed to the detailed CC-practice section, whereas those who answered “No” completed the shorter non-user section. All items were mandatory, and no partial questionnaires could be submitted. Respondents were able to review and revise their answers before final submission; however, responses could not be modified after submission. The platform recorded submission timestamps, but individual completion time was not automatically measured.

The study was reported following the STROBE guidelines, and key methodological aspects of the CHERRIES checklist for internet-based surveys were considered [24,25].

### 2.4. Statistical Analysis

Statistical analyses were performed using IBM SPSS Statistics for Windows, version 31.0 (IBM Corp., Armonk, NY, USA). The normality of continuous variables was assessed using both visual methods (histograms and probability plots) and analytical tests.

Categorical variables were analyzed using the chi-square test or Fisher’s exact test, as appropriate, with Bonferroni correction applied for post hoc comparisons. Continuous variables were compared with the Mann–Whitney U test. Multiple-response items were evaluated as response counts rather than participant counts.

To identify factors independently associated with CC use, multivariable logistic regression analysis was conducted. Variables that were statistically significant in univariable analysis and considered clinically relevant were included in the multivariable logistic regression model. Due to potential collinearity between age and years in practice, only years as a urology specialist were retained in the final model to represent professional seniority. Collinearity diagnostics showed no evidence of problematic multicollinearity among the variables retained in the final model. VIF values were 1.168 for years as a urology specialist, 1.223 for type of institution, 1.072 for weekly new male infertility case volume, and 1.120 for andrology-focused practice. Results are reported as odds ratios (ORs) with 95% confidence intervals (CIs). Model calibration was assessed using the Hosmer–Lemeshow goodness-of-fit test, and classification performance was evaluated using overall accuracy, sensitivity, and specificity. Model performance was further evaluated using the area under the receiver operating characteristic curve (AUC), Cox & Snell R^2^, and Nagelkerke R^2^. The cutoffs for years as a urology specialist (>20 vs. ≤20 years) and weekly new male infertility cases (>5 vs. ≤5 cases/week) were based on predefined questionnaire categories, clinical interpretability, and the need to reduce sparse cells in multivariable modeling. Because dichotomization may reduce information, sensitivity analyses were performed using the original ordinal categories for years as a specialist and weekly new male infertility case volume.

A two-sided *p*-value less than 0.05 was considered statistically significant.

## 3. Results

### 3.1. Participant Demographics and Professional Characteristics

A total of 402 urologists participated in the survey, of whom 158 (39.3%) reported using CC in their clinical practice, whereas 244 (60.7%) reported no use. CC users were significantly older than non-users (median age 46.5 vs. 41.0 years, *p* < 0.001), whereas gender and academic degree were not associated with CC use. In univariate analysis, professional and practice-related factors, including professional seniority, institutional setting, subspecialty, particularly andrology, and clinical exposure to male infertility, were associated with CC use. These demographic and professional characteristics are shown in Table 1.

In multivariable logistic regression analysis (Table 2), working in a private practice setting was independently associated with a higher likelihood of CC use compared with non-private settings (OR = 2.90, 95% CI = 1.70–4.93, *p* < 0.001). Similarly, urologists with more than 20 years of experience were more likely to prescribe CC than those with 20 years or less of experience (OR = 2.18, 95% CI = 1.34–3.56, *p* = 0.002). A higher infertility case volume, defined as more than five new cases per week, was also independently associated with increased CC use (OR = 2.27, 95% CI = 1.41–3.67, *p* = 0.001). Although an andrology-focused practice showed a higher likelihood of CC use, this association did not reach statistical significance (OR = 2.35, 95% CI = 0.85–6.49, *p* = 0.098).

The model showed no evidence of poor fit on the Hosmer–Lemeshow test (χ^2^ = 6.190, *p* = 0.185), with Cox & Snell R^2^ = 0.129 and Nagelkerke R^2^ = 0.175. The AUC was 0.779 (95% CI: 0.734–0.824, *p* < 0.001). Classification analysis showed an overall accuracy of 67.9%, specificity of 86.1%, and sensitivity of 39.9%, indicating that the model was better at identifying non-users than CC users at the default 0.50 cutoff. Sensitivity analyses using the original ordinal categories for years as a specialist and weekly new male infertility case volume showed broadly consistent findings with the primary model. Type of institution remained significantly associated with CC use (*p* = 0.001), andrology-focused practice remained non-significant (*p* = 0.142), and both years as a specialist and weekly new male infertility case volume remained significantly associated with CC use as overall categorical variables (*p* = 0.001 and *p* = 0.004, respectively).

### 3.2. Reasons for Non-Use of Clomiphene Citrate

Among urologists who did not prescribe CC, several reasons for non-use were reported, including insufficient evidence or uncertainty about effectiveness, perceived inconsistency with current clinical guidelines, uncertainty about patient selection, lack of knowledge or clinical experience, and a preference for alternative treatments or referral to assisted reproductive technology (ART) as a first-line approach. All reported reasons for non-use are summarized in Table 3.

### 3.3. Indications, Contraindications, and Treatment Goals

Among CC users, idiopathic male infertility was the most commonly reported indication (77.2%), followed by hypogonadotropic hypogonadism and unexplained infertility. In contrast, hypergonadotropic hypogonadism was rarely selected as an indication (10.8%) but was the most frequently cited contraindication (78.5%). Treatment goals were primarily focused on laboratory outcomes, with improvements in sperm concentration and increases in serum testosterone levels being the most commonly reported objectives. Goals related to sperm motility, pregnancy or live birth rate, morphology, and sexual function were reported less frequently. Indications, contraindications, and treatment goals of CC use are shown in Table 4.

### 3.4. Pretreatment Evaluation and Treatment Strategies

Pretreatment evaluation primarily relied on standard assessments. Most respondents routinely performed semen analysis and baseline hormonal tests, including total testosterone, follicle-stimulating hormone (FSH), and luteinizing hormone (LH). Estradiol measurement and physical examination were also commonly reported, whereas advanced tests, such as sperm DNA fragmentation testing and dual-energy X-ray absorptiometry, were used less frequently. Substantial variation was observed in CC dosing strategies, with daily doses of 25 mg or 50 mg being the most common, followed by cyclic regimens and alternate-day schedules. High-dose regimens of 100 mg/day or higher were rarely reported. Most urologists prescribed CC together with antioxidants or nutritional supplements, whereas CC monotherapy or combination therapy with other hormonal agents was less frequently reported. Pretreatment evaluation and treatment strategies are shown in Table 5.

### 3.5. Risk Counseling and Follow-Up Practices

Reported risk counseling practices varied widely. Before starting CC therapy, counseling most often focused on possible increases in liver enzymes (41.8%), gynecomastia (36.1%), and changes in libido (35.4%), whereas counseling about thromboembolic risk, mood changes, headache, visual disturbances, deterioration of semen parameters, and bone mineral density loss was less common. Notably, 31.6% of respondents reported not providing routine risk counseling before treatment initiation. Follow-up strategies mainly focused on hormonal levels and physical examination findings. During the first follow-up visit, total testosterone, FSH, and LH were assessed more frequently than semen analysis or advanced semen testing. Follow-up visits were most often scheduled every three months. The typical duration of CC therapy ranged from 4 to 6 months, followed by 3 months; longer treatment durations were less frequently reported. Risk counseling and follow-up practices are shown in Table 6.

### 3.6. Treatment Success Definitions and Management Strategies

Definitions of treatment success varied widely among respondents. Improvement in semen parameters was the most frequently reported indicator of success, followed by spontaneous pregnancy, increased success rates with ART, and increases in serum testosterone levels, whereas symptom improvement was reported less often. Thus, although surrogate laboratory markers predominated, pregnancy-related outcomes were also commonly included in perceived definitions of treatment success. Live birth was not separately assessed as an independent outcome in this survey. Among patients perceived as responders at three months, the most commonly preferred strategy was to continue treatment at the same dose for up to six months (60.8%). In contrast, management of non-responders most frequently involved referral for ART (70.3%), followed by the addition of antioxidants or supplements, continuation of current treatment with reassessment, or switching to another empirical medical therapy. Treatment success definitions and management strategies are shown in Table 7.

### 3.7. Perceived Success Rates and Adverse Effects

**Figure 2:**
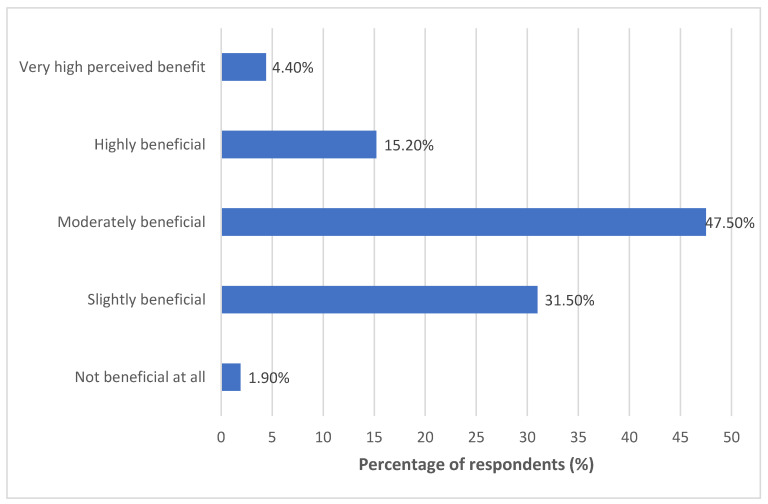
Respondents’ self-reported evaluation of fertility outcomes associated with clomiphene citrate use. Most respondents rated CC as moderately beneficial (47.5%) or slightly beneficial (31.0%), whereas fewer reported high (15.2%) or very high perceived benefit (4.4%). Only 1.9% rated it as not beneficial at all.

Self-reported success rates varied across different outcome measures. Based on respondents’ definitions of treatment success, the perceived clinical benefit of CC was most often rated as moderate or slight (Figure 2).

Although various adverse effects were reported, including changes in libido, gastrointestinal symptoms, and elevated liver enzymes, frequent or severe adverse events were rare. Observed adverse effects during CC therapy are shown in Table 8.

When adverse effects occurred, preferred management strategies included discontinuing treatment (37.3%), temporarily stopping treatment (18.4%), and lowering the dose (16.5%). Conversely, 25.3% of respondents reported no adverse effects requiring treatment modification.

## 4. Discussion

### 4.1. Principal Findings

This survey captures how urologists navigate CC prescribing in male infertility amid limited evidence and cautious guideline recommendations. Instead of assessing efficacy, the study focuses on real-world decision-making within a therapeutic “gray zone,” where practice reflects adapting to uncertainty rather than following evidence-based consensus.

Only two in five respondents reported using CC, showing that its use remains limited even within a single urology community in Türkiye. Non-use mainly stemmed from concerns about insufficient evidence, inconsistent with guidelines, and uncertainty regarding patient selection. However, because participation was voluntary, this proportion should be interpreted as the rate of CC use among respondents rather than as an estimate of CC prescribing prevalence among all urologists in Türkiye.

CC prescribing was not evenly distributed across respondent subgroups. Multivariable analysis showed that clinician seniority, private-sector practice, and higher infertility case volume were independently associated with CC use. Although andrology-focused practice was associated with higher CC use in univariable analysis, this association did not remain statistically significant after adjustment, possibly due to limited power. Overall, these findings suggest that CC prescribing patterns are mainly associated with clinician experience and practice setting rather than standardized protocols.

Importantly, treatment goals and perceived definitions of success were mainly based on surrogate laboratory endpoints, including semen parameters and hormonal changes, whereas patient-centered reproductive outcomes were not directly measured at the patient level in this survey; however, spontaneous pregnancy was commonly reported as a perceived success definition, and live birth was not separately assessed.

Taken together, these findings indicate that CC prescribing among responding urologists is largely shaped by clinician experience, practice setting, and surrogate biological markers rather than standardized prescribing and outcome-assessment frameworks.

### 4.2. Navigating the Evidence-Guideline Gap: The Role of Experience

The variability in CC use likely reflects an adaptive, experience-based response to the lack of consensus-driven clinical guidance. Current international guidelines emphasize the low quality and inconsistency of evidence supporting selective estrogen receptor modulators in male infertility and therefore refrain from making strong recommendations [4,8]. In this context, our findings suggest that clinicians frequently extend beyond these cautious guideline positions in real-world practice—particularly in cases of idiopathic infertility—indicating a clear divergence between guideline recommendations and clinical decision-making, and highlighting the need for more robust evidence and better alignment between research, guidelines, and practice [4,8]. While certain aspects of practice, such as avoidance in hypergonadotropic hypogonadism, appear to align with guideline principles, the frequent use of CC in idiopathic infertility and heterogeneous dosing strategies reflect areas of divergence from current recommendations.

This experience-driven pattern aligns with prior research. Thaker et al. reported that about 80% of respondents used empirical medical therapies, with nearly all CC use among reproductive urologists (98%) [17]. Similarly, Catford et al. found that CC was the most frequently selected agent (78%) among EMT users, even though urologists accounted for only a minority of respondents [18]. In our study, CC use was also more common among clinicians with higher infertility case volume, private-sector practice, and greater years of professional experience, further supporting the influence of clinical autonomy and experience on prescribing behaviors.

Taken together, these findings suggest that, in the absence of definitive evidence, prescribing decisions appear to rely more on clinician judgment and contextual experience than on formal evidence hierarchies, reflecting a shift toward experience- and context-based decision-making in male infertility management.

Notably, this pattern does not seem to be exclusive to CC. Both earlier and recent survey data indicate that empirical medical treatments for idiopathic male infertility often include other EMTs, especially hCG/LH, aromatase inhibitors, and, in certain cases, FSH-based regimens. There is considerable variation among clinicians in terms of medication choices and adoption rates [17,18,19]. In previous AUA survey literature, the most frequently used agents were clomiphene citrate, hCG, and anastrozole. More recent research continues to show clomiphene and hCG as common options, despite the lack of an officially approved medical therapy for idiopathic male infertility [17,18,19].

This broader EMT landscape highlights the same structural tensions found in our study: widespread real-world application, dependence on clinician judgment, and ongoing reliance on surrogate endpoints—like hormonal or semen parameters—in a field where guidelines are cautious and clinically significant outcomes, such as pregnancy and live birth, are still not well-established [4,8,15,16].

Viewed in this context, the gap between evidence and practice for CC should not be seen as an isolated prescribing issue but rather as a broader characteristic across the field of empirical medical therapy in male infertility. This has important implications for research and clinical practice, highlighting the need for standardized outcome frameworks, greater emphasis on patient-centered endpoints, and more consistent integration of evidence into clinical decision-making.

Importantly, although this study was conducted within a national context, the observed variability in clomiphene citrate prescribing is unlikely to be unique to Türkiye. Similar patterns of heterogeneity in empirical medical therapy use have been reported in national surveys from other regions, including North America and Australia, where clinician experience and local practice environments similarly shape treatment decisions [17,18,19].

Therefore, our findings should be interpreted not only as a description of practices among responding urologists in Türkiye but also as an illustrative example of broader challenges in the empirical management of male infertility.

### 4.3. Surrogate Endpoints and Reproductive Outcomes

The reliance on surrogate outcomes in our study underscores a well-known limitation in the male infertility field. A systematic review of randomized controlled trials has revealed significant discrepancies in outcome reporting, mainly emphasizing semen-related measures and often overlooking clinically meaningful endpoints such as pregnancy and live birth [15].

In our survey, surrogate laboratory endpoints were the most frequently reported measures of treatment goals and perceived success; however, this should not be interpreted as an absence of reproductive goals in clinical practice. Spontaneous pregnancy was selected as a success definition by 66.5% of CC users, indicating that pregnancy-related outcomes remained common. Nevertheless, pregnancy represents an intermediate reproductive endpoint, whereas live birth is a final reproductive endpoint; live birth was not separately assessed in this survey.

Although meta-analyses suggest that CC and other hormonal therapies may improve semen parameters and hormonal profiles [12,16,26,27,28], the extent to which these biological changes translate into reproductive outcomes remains uncertain. This issue is also supported by earlier randomized evidence, including the World Health Organization trial, which found no significant increase in pregnancy rates despite hormonal treatment [29].

These findings collectively highlight a critical gap between surrogate markers and patient-centered outcomes, indicating that clinical decisions are often influenced more by biological signals than by robust evidence of reproductive success. This gap is further complicated by the fact that outcomes such as pregnancy and live birth reflect the integrated contribution of both partners and the broader reproductive context, making it difficult to attribute these endpoints solely to male-directed therapy in real-world settings.

A more structured approach to outcome assessment may therefore be achieved through a tiered framework, incorporating (1) male-biologic endpoints (e.g., semen parameters and hormonal profiles), (2) intermediate couple-level outcomes such as time-to-pregnancy and clinically confirmed pregnancy, and (3) ultimate reproductive outcomes such as live birth. Such a framework may help balance clinical relevance with feasibility while providing a more comprehensive evaluation of treatment effects.

Future research should accordingly prioritize pragmatic study designs and multicenter registries that capture both biological and reproductive outcomes while explicitly accounting for female factors and shared decision-making processes. In addition, the development of core outcome sets incorporating clinically meaningful endpoints—such as time-to-pregnancy, pregnancy, and live birth—may improve comparability across studies and enhance the clinical relevance of emerging evidence.

Emerging international frameworks—such as the APHRODITE classification—further aim to standardize patient stratification by grouping men with similar clinical and biological profiles. By reducing heterogeneity in study populations, these approaches may enable more consistent evaluation of treatment effects and support the development of evidence-based, outcome-oriented clinical guidelines for male infertility [30].

### 4.4. Patient Selection and the Indication Paradox

In Türkiye, the evaluation and management of male infertility may often be performed by general urologists rather than within a uniformly structured andrology subspecialty framework. Although European guidelines recommend that the male partner be evaluated by a urologist trained in male reproductive health [4], access to standardized andrology-focused training and referral pathways may vary across clinical settings. In practice, most clinicians manage male infertility based on residency training, sometimes supplemented by short-term courses or fellowships. This organizational structure may partially explain the heterogeneity observed in clinical practice patterns. Similar training models are reported in other countries, where male reproductive health is incorporated into urology residency with optional subspecialty training opportunities [19].

Within this clinical and educational context, substantial heterogeneity in patient selection was observed. While CC is commonly used for idiopathic infertility and hypogonadotropic hypogonadism, the latter was also identified as a contraindication by a notable proportion of respondents, suggesting an apparent “indication paradox.” However, this finding should be interpreted cautiously because the questionnaire did not distinguish between different forms of hypogonadotropic hypogonadism, such as functional, reversible, congenital, pituitary-related, or irreversible forms. Therefore, this pattern may reflect differing interpretations of the clinical scenario and terminology rather than a true contradiction in clinical reasoning or diagnostic misunderstanding among respondents.

Men with functional or reversible hypogonadism and preserved pituitary reserve may benefit from CC, whereas those with irreversible hypogonadism usually require exogenous gonadotropins [31]. Baseline gonadotropin levels, especially follicle-stimulating hormone (FSH), are important predictors of treatment response, with low to normal levels generally linked to better outcomes [31]. Conversely, hypergonadotropic hypogonadism correctly indicates primary testicular failure and is a contraindication.

Importantly, the coexistence of hypogonadotropic hypogonadism as both an indication and a contraindication may reflect ambiguity in terminology and heterogeneity in clinical stratification. This may reflect variable terminology and heterogeneous clinical stratification rather than a clearly demonstrable knowledge gap. Consequently, this apparent “indication paradox” should be interpreted as a possible signal of variable terminology and patient stratification rather than as definitive evidence of a knowledge gap, emphasizing the need for clearer definitions, standardized thresholds, and evidence-based frameworks for patient selection. In this context, a simplified clinical stratification framework based on baseline gonadotropin levels and clinical context may help reduce ambiguity, minimize misclassification and improve consistency in clinical decision-making.

### 4.5. Dosing, Monitoring, and Empirical Pragmatism

Our findings demonstrate substantial heterogeneity in CC dosing, treatment duration, and monitoring practices, reflecting empirical pragmatism rather than standardized protocols. Reported regimens ranged from 25–50 mg daily to alternate-day and cyclic schedules, consistent with existing literature [9,12].

Treatment duration generally ranged from 3–6 months, although shorter (8–12 weeks) and longer (6–12 months) protocols have been documented in experimental and observational studies [32]. In our group, most clinicians reported treatment durations of 4–6 months, with follow-up intervals usually set at 8–12 weeks. Monitoring methods were mainly laboratory-based, focusing on serum testosterone, LH, FSH, and semen parameters [32].

In the absence of robust comparative evidence linking specific protocols to clinically meaningful fertility outcomes, this heterogeneity likely represents an adaptive response rather than inconsistent practice. However, it also limits comparability across studies and highlights the need for standardized treatment pathways.

### 4.6. Safety Perceptions and Gaps in Informed Consent

Consistent with previous reports, CC was generally perceived as safe and well-tolerated, with serious adverse events reported infrequently [12,33]. However, nearly one-third of clinicians did not routinely provide formal risk counseling before treatment, representing a notable gap in clinical practice. Although often regarded as a harmless EMT, CC carries potential risks. A systematic review reported paradoxical worsening in semen parameters in some men, with rates between 17% and 24% [34]. Additional rare but clinically relevant complications, including visual disturbances, have been reported; evidence on thromboembolic risk remains limited and should be framed cautiously [35,36]. Possible negative effects on bone mineral density have also been described [37], further suggesting that this therapy is not without risks.

Given that 31.6% of respondents reported no routine risk counseling, and considering the off-label use of CC for male infertility, structured pre-treatment counseling should be considered a minimum standard of responsible prescribing. This is particularly important because consistent evidence linking improvements in hormonal or semen parameters to clinically meaningful reproductive outcomes remains limited, including pregnancy as an intermediate endpoint and live birth as a final endpoint [15,16,38,39]. At a minimum, counseling should include discussion of: (1) the off-label status of CC for male infertility; (2) the uncertainty regarding its effect on reproductive outcomes, including pregnancy and live birth as distinct endpoints; (3) the possibility that improvements in semen parameters or hormonal profiles may not translate into reproductive benefit; (4) the potential for paradoxical worsening of semen parameters; (5) less frequent but clinically relevant risks, including visual disturbances and thromboembolic complications; (6) common or monitorable adverse effects, such as mood changes, libido changes, gynecomastia, gastrointestinal symptoms, liver enzyme elevation, and possible bone mineral density effects; and (7) the planned monitoring strategy, treatment duration, and stopping criteria [12,33,34,35,36,37,38,39].

In addition, documenting this counseling in the medical record—including the clinical rationale, uncertainties discussed, and planned follow-up—may support both shared decision-making and medico-legal transparency, particularly in the context of off-label empirical therapy [38]. 

Taken together, these findings underscore the importance of structured counseling and shared decision-making in managing male infertility. Clinicians should clearly communicate both the potential benefits and limitations of CC therapy as part of responsible prescribing. These results should be interpreted cautiously, as self-reported practices may be influenced by social desirability bias.

### 4.7. Strengths, Limitations, and Clinical Implications

In light of these findings, the present study demonstrates that the challenges observed are not confined to a single national context but reflect a broader, field-wide issue in male infertility management. Addressing this variability will require coordinated international efforts to standardize definitions, harmonize treatment protocols, and prioritize clinically meaningful reproductive outcomes.

This study has several strengths, including its nationwide recruitment strategy, a relatively large sample size compared with many physician surveys, and the use of multivariable modeling to identify factors independently associated with CC use.

Several limitations should be recognized. First, although the sample size was relatively large, the participation rate (27%) may be considered modest and should be interpreted with caution. Physician web-based surveys among specialists typically yield comparable response rates, supporting the methodological acceptability of our design but not excluding the possibility of non-response bias [40,41,42]. Because participation was voluntary and the survey was distributed through professional WhatsApp groups, respondents may represent a subgroup of urologists with greater interest, experience, or clinical exposure to male infertility and empirical medical therapy. This may have led to an overestimation of both CC use and perceived benefit if clinicians with more favorable experiences or greater interest in CC were more likely to participate.

Second, a formal comparison with demographic data from the Society of Urological Surgery in Türkiye could not be performed, as publicly available sources lack sufficiently detailed member-level characteristics, such as age, practice sector, subspecialty profile, and infertility caseload. Consequently, the characteristics of non-responders remain unknown, limiting the ability to formally assess representativeness or apply weighted adjustments. Therefore, the findings should be interpreted as reflecting the practices of participating urologists rather than those of all urologists in Türkiye.

Third, reliance on self-reported practices introduces the possibility of recall bias and social desirability bias. Fourth, although the questionnaire was developed using guideline- and literature-based input, expert consensus, and pilot testing, it was not formally psychometrically validated. Therefore, findings related to perceived benefit, counseling quality, and treatment success definitions should be interpreted as descriptive self-reported practice patterns rather than as validated measures of underlying attitudes, knowledge, or quality of care. Another limitation is that the questionnaire did not collect respondents’ city or geographical region of practice. Therefore, although the survey invitation was distributed through national and regional professional WhatsApp groups covering all seven geographical regions of Türkiye, the geographic distribution of respondents could not be analyzed. Accordingly, the national scope of the study should be interpreted as reflecting nationwide recruitment rather than formal geographic representativeness. Future studies should consider formal psychometric validation of survey instruments to better assess these constructs.

Finally, the study does not include patient-level outcomes and therefore cannot evaluate the effectiveness of CC on clinically meaningful fertility endpoints. In addition, multivariable analysis was limited to available clinician-level variables, and residual confounding may still be present.

Despite these limitations, the findings provide valuable insights into how clinicians handle therapeutic uncertainty and highlight areas with notable practice variation. Three main priorities emerge: (1) establishing consensus-based patient selection criteria, including standardized interpretation of gonadotropins and baseline semen parameters; (2) setting minimum standards for monitoring and patient counseling, particularly emphasizing the gap between surrogate improvements and actual fertility outcomes; and (3) aligning future research with patient-centered endpoints and standardized outcome definitions. Multicenter prospective studies and registries that include both biological and reproductive outcomes, such as time-to-pregnancy and live birth, are crucial for bridging the gap between surrogate markers and meaningful clinical outcomes. Additionally, creating core outcome sets for male infertility trials would improve comparability and increase the relevance of emerging evidence.

## 5. Conclusions

Clomiphene citrate continues to be used in male infertility despite uncertainty regarding its clinical benefit and considerable variability in real-world practice. In this national cross-sectional survey among responding urologists, CC prescribing was mainly associated with clinician experience, practice setting, and infertility case volume rather than standardized protocols. Treatment goals and perceived success definitions were most frequently based on surrogate laboratory endpoints, including semen parameters and hormonal changes, although spontaneous pregnancy was also commonly reported as a perceived success definition. Live birth was not separately assessed. The observed heterogeneity in patient selection, dosing, monitoring, counseling, and success definitions, together with the apparent indication paradox for hypogonadotropic hypogonadism, which may reflect differing interpretations of functional versus irreversible hypogonadotropic states, highlights the need for clearer terminology, standardized prescribing frameworks, and future studies incorporating clinically meaningful reproductive endpoints.

## Figures and Tables

**Table 1:** Demographic and professional characteristics of urologists according to clomiphene citrate use.

Variable	Category	Yes (*n*, %)	No (*n*, %)	*p* Value
Age (continuous)	Median (IQR)	46.5 (16.0)	41.0 (13.0)	<0.001 ^y^
Gender	Male	158 (100)	242 (99.2)	0.522
	Female	0 (0.0)	2 (0.8)	
Age group (years)	30–39	40 (25.3) ^a^	105 (43.0) ^b^	<0.001 ^χ^
	40–49	53 (33.5)	83 (34.0)	
	50–59	36 (22.8) ^a^	34 (13.9) ^b^	
	60–69	23 (14.6) ^a^	20 (8.2) ^b^	
	70–79	6 (3.8) ^a^	2 (0.8) ^b^	
Academic degree	Specialist	89 (56.3)	144 (59.0)	0.658
	Assistant Professor	17 (10.8)	30 (12.3)	
	Associate Professor	31 (19.6)	47 (19.3)	
	Professor	21 (13.3)	23 (9.4)	
Years as a urology specialist	<5 years	16 (10.1) ^a^	73 (29.9) ^b^	0.001 ^χ^
	5–10 years	28 (17.7)	47 (19.3)	
	11–20 years	46 (29.1)	73 (29.9)	
	>20 years	68 (43.0) ^a^	51 (20.9)ᵇ	
Type of institution	State hospital	33 (20.9)	60 (24.6)	0.001 ^χ^
	Training & research/City hospital	30 (19.0) ^a^	111 (45.5) ^b^	
	University hospital	34 (21.5)	37 (15.2)	
	Private hospital/clinic	61 (38.6) ^a^	36 (14.8) ^b^	
Main urology subspecialty	General urology	130 (82.3)	191 (78.3)	<0.001 ^χ^
	Andrology	18 (11.4) ^a^	6 (2.5) ^b^	
	Uro-oncology	4 (2.5) ^a^	20 (8.2) ^b^	
	Endourology	5 (3.2) ^a^	20 (8.2) ^b^	
	Functional urology	1 (0.6)	3 (1.2)	
	Pediatric urology	0 (0.0)	4 (1.6)	
Infertility patient proportion	None	1 (0.6)	2 (0.8)	<0.001 ^χ^
	<10%	92 (58.2) ^a^	192 (78.7) ^b^	
	11–25%	47 (29.7) ^a^	45 (18.4) ^b^	
	26–50%	11 (7.0) ^a^	5 (2.0) ^b^	
	51–75%	2 (1.3)	0 (0.0)	
	>75%	5 (3.2) ^a^	0 (0.0) ^b^	
Weekly new male infertility cases	None	1 (0.6)	8 (3.3)	0.004 ^χ^
	1–5	97 (61.4) ^a^	177 (72.5) ^b^	
	6–10	37 (23.4)	45 (18.4)	
	11–20	20 (12.7) ^a^	10 (4.1) ^b^	
	21–50	3 (1.9)	4 (1.6)	

Values are presented as *n* (%) unless otherwise indicated. Continuous variables are expressed as median (IQR). Superscript letters (^a^, ^b^) denote statistically significant pairwise comparisons within each variable; categories sharing the same letter are not significantly different, whereas those with different letters differ significantly (Bonferroni-adjusted). ^χ^ Pearson chi-square test; ^y^ Mann–Whitney U test. Statistical significance was set at *p* < 0.05.

**Table 2:** Multivariable logistic regression analysis of factors associated with CC use, including model fit statistics.

Variable	OR	95% CI	*p*-Value
>20 years of experience	2.18	1.34–3.56	0.002
Private practice	2.90	1.70–4.93	<0.001
Andrology practice	2.35	0.85–6.49	0.098
>5 cases/week	2.27	1.41–3.67	0.001

OR, odds ratio; CI, confidence interval. Reference categories: ≤20 years as a urology specialist; non-private practice setting; non-andrology subspecialty; ≤5 new male infertility cases per week. Variables entered into the model: years as a urology specialist (>20 vs. ≤20 years), type of institution (private vs. non-private), urology subspecialty (andrology vs. non-andrology), and weekly number of new male infertility cases (>5 vs. ≤5).

**Table 3:** Reported reasons for non-use of clomiphene citrate among non-users.

Reason for Non-Use	*n* (Responses)	% of Respondents *
Insufficient evidence/uncertain efficacy	97	39.8
Inconsistency with clinical guidelines	90	36.9
Uncertainty in patient selection	72	29.5
Lack of knowledge or experience	70	28.7
Preference for alternative treatments	56	23.0
Referral to ART as the first-line approach	55	22.5
Lack of benefit in personal clinical experience	43	17.6
Concerns about adverse effects	37	15.2
Medico-legal concerns	30	12.3
Lack of FDA approval	22	9.0
Laboratory and follow-up limitations	20	8.2
Risk of paradoxical deterioration in semen parameters	16	6.6
Drug availability or cost issues	16	6.6
Referral to endocrinology	16	6.6
Patient preference or poor compliance	15	6.1
Concerns regarding bone health/monitoring burden	3	1.2
Other	9	3.7
Total responses	667	273.4

Multiple responses allowed. ART, assisted reproductive technology; FDA, U.S. Food and Drug Administration. * Percentage calculated based on the number of non-users (*n* = 244).

**Table 4:** Indications, contraindications, and treatment goals of clomiphene citrate.

Domain	Item	*n*	% of Respondents *
Indications	Idiopathic male infertility	122	77.2
	Hypogonadotropic hypogonadism	75	47.5
	Unexplained infertility	67	42.4
	Hypergonadotropic hypogonadism	17	10.8
	Other	7	4.4
Contraindications	Hypergonadotropic hypogonadism	124	78.5
	Hypogonadotropic hypogonadism	42	26.6
	Unexplained infertility	22	13.9
	Idiopathic male infertility	10	6.3
	Other	2	1.3
Primary treatment goals	Increase sperm count	134	84.8
	Increase serum testosterone	101	63.9
	Improve sperm motility	66	41.8
	Increase pregnancy or live birth rate	65	41.1
	Improve morphology	44	27.8
	Improve libido/sexual function	44	27.8

Multiple responses allowed. * Percentage calculated based on the number of users (*n* = 158). The treatment-goal item “Increase pregnancy or live birth rate” was presented as a single combined questionnaire option; pregnancy and live birth were not assessed separately in this item.

**Table 5:** Pretreatment assessment, dosing regimens, and treatment modalities among clomiphene citrate users.

Category	Parameter/Strategy	*n*	%
Pretreatment assessment	Semen analysis	141	89.2
	Total testosterone	138	87.3
	FSH	138	87.3
	LH	121	76.6
	Estradiol	90	57.0
	Physical examination	88	55.7
	Prolactin	71	44.9
	Liver function tests	71	44.9
	Erectile function evaluation	63	39.9
	Free testosterone	55	34.8
	Scrotal ultrasonography	55	34.8
	Sperm DNA fragmentation/advanced tests	19	12.0
	DEXA scan	1	0.6
Dosing regimen	25 mg/day	44	27.8
	50 mg/day	37	23.4
	50 mg/day (25 on/5 off)	34	21.5
	25 mg (25 on/5 off)	23	14.6
	50 mg every other day	14	8.9
	≥100 mg/day	2	1.3
	Other/individualized regimen	4	2.5
Treatment modality	CC and antioxidants/supplements	127	80.4
	CC monotherapy	37	23.4
	CC and aromatase inhibitor	22	13.9
	CC and gonadotropins	18	11.4

Multiple responses allowed. Percentage calculated based on the number of users (*n* = 158).

**Table 6:** Reported adverse-effect counseling, follow-up assessments, timing, and treatment duration among clomiphene citrate users.

Domain	Item	*n*	%
Risks routinely discussed	Elevated liver enzymes	66	41.8
	Gynecomastia	57	36.1
	Libido changes	56	35.4
	Thromboembolic complications	51	32.3
	Mood changes/irritability	42	26.6
	Headache	33	20.9
	Visual disturbances	30	19.0
	Sperm deterioration	24	15.2
	Bone mineral density loss	18	11.4
	No routine counseling	50	31.6
Parameters at first follow-up	Total testosterone	119	75.3
	Physical examination	111	70.3
	FSH	109	69.0
	LH	94	59.5
	Estradiol	64	40.5
	Liver function tests	66	41.8
	Semen analysis	32	20.3
	Advanced semen tests	5	3.2
Follow-up timing	Monthly	34	21.5
	Every 3 months	114	72.2
	Every 6 months	7	4.4
	No routine follow-up	2	1.3
Typical treatment duration	4–6 months	94	59.5
	3 months	32	20.3
	7–9 months	15	9.5
	10–12 months	12	7.6

Multiple responses were allowed for specific counseling topics. ‘No routine counseling’ was treated as a mutually exclusive response category. Percentages were calculated based on the number of CC users (*n* = 158).

**Table 7:** Reported definitions of treatment success and management approaches for responders and non-responders among clomiphene citrate users.

Domain	Definition/Strategy	*n*	%
Definition of success	Semen parameter improvement	149	94.3
	Spontaneous pregnancy	105	66.5
	Increased ART success	74	46.8
	Serum testosterone increases	77	48.7
	Symptom improvement	52	32.9
Management of responders at 3 months	Continue the same dose up to 6 months	96	60.8
	Discontinue treatment	27	17.1
	Gradually taper the dose	14	8.9
	Continue up to 12 months	14	8.9
	Introduce drug holidays at defined intervals	15	9.5
	Continue treatment until pregnancy is achieved	11	7.0
Management of non-responders at 3 months	Refer to ART	111	70.3
	Add antioxidants/supplements	54	34.2
	Continue current treatment and reassess after 3 months	33	20.9
	Switch to another empirical therapy	35	22.2
	Add gonadotropins (FSH or hMG)	31	19.6
	Add an aromatase inhibitor to the current treatment	20	12.7
	Increase the dose of the current treatment	11	7.0

Multiple responses allowed. Percentage calculated based on the number of users (*n* = 158).

**Table 8:** Self-reported frequency of adverse effects during clomiphene citrate therapy among CC users.

Adverse Effect	None, *n* (%)	Rare, *n* (%)	Occasional, *n* (%)	Frequent, *n* (%)	Undecided, *n* (%)
Visual disturbances	93 (58.9)	51 (32.3)	8 (5.1)	2 (1.3)	4 (2.5)
Mood changes/irritability	54 (34.2)	71 (44.9)	21 (13.3)	5 (3.2)	7 (4.4)
Changes in libido	46 (29.1)	62 (39.2)	38 (24.1)	7 (4.4)	5 (3.2)
Gastrointestinal complaints	53 (33.5)	79 (50)	18 (11.4)	4 (2.5)	4 (2.5)
Elevated liver enzymes	52 (32.9)	75 (47.5)	21 (13.3)	4 (2.5)	6 (3.8)
Deterioration in semen parameters	54 (34.2)	76 (48.1)	21 (13.3)	4 (2.5)	3 (1.9)
Headache/dizziness	62 (39.2)	62 (39.2)	24 (15.2)	6 (3.8)	4 (2.5)
Decrease in bone mineral density	86 (54.4)	56 (35.4)	5 (3.2)	1 (0.6)	10 (6.3)

Note: CC, clomiphene citrate. Percentages were calculated based on the number of CC users (*n* = 158). Frequency categories reflect respondents’ self-reported observations during CC therapy. Rare, occasional, and frequent adverse effects were defined as <1%, 1–5%, and >5%, respectively. Percentages may not total 100% exactly because of rounding.

## Data Availability

The datasets generated during this study are not publicly accessible due to participant confidentiality, but can be obtained from the corresponding author upon reasonable request.

## Abbreviations

The following abbreviations are used in this manuscript:

APHRODITE	Addressing Male Patients with Hypogonadism and/or Infertility Owing to Altered Idiopathic Testicular Function
ART	Assisted reproductive technology
AUA	American Urological Association
CC	Clomiphene citrate
CHERRIES	Checklist for Reporting Results of Internet E-Surveys
CI	Confidence interval
DEXA	Dual-energy X-ray absorptiometry
DNA	Deoxyribonucleic acid
EAU	European Association of Urology
EMT	Empirical medical therapy
FDA	Food and Drug Administration
FSH	Follicle-stimulating hormone
hCG	Human chorionic gonadotropin
hMG	Human menopausal gonadotropin
IQR	Interquartile range
LH	Luteinizing hormone
OR	Odds ratio
SERM	Selective estrogen receptor modulator
SPSS	Statistical Package for the Social Sciences
STROBE	Strengthening the Reporting of Observational Studies in Epidemiology
